# Association between Health Practice and Food Stockpiling for Disaster

**DOI:** 10.3390/nu13051414

**Published:** 2021-04-23

**Authors:** Moeka Harada, Rie Kobayashi, Jun Oka, Nobuyo Tsuboyama-Kasaoka

**Affiliations:** 1 Department of Food and Nutrition, Faculty of Home Economics, Tokyo Kasei University, Tokyo 173-8602, Japan; harada-mo@tokyo-kasei.ac.jp; 2 Section of Global Disaster Nutrition, International Center for Nutrition and Information, National Institute of Health and Nutrition, National Institutes of Biomedical Innovation, Health and Nutrition, Tokyo 162-8636, Japan; ntsubo@nibiohn.go.jp; 3 Department of Rehabilitation, Faculty of Health Sciences, Tokyo Kasei University, Saitama 350-1398, Japan; okajun@tokyo-kasei.ac.jp

**Keywords:** food stockpiling, food for disaster, disaster preparedness, healthy lifestyle, health practice

## Abstract

In this study investigated the association between health practices and food stockpiling for disasters in predicted areas with a high risk of food shortage due to the Nankai Trough earthquake. A survey was conducted during 18–20 December 2019 using a self-administered web-based questionnaire. In total, 1200 individuals registered with an online survey company participated in the study. The association between health practices and food stockpiling status was analyzed (*n* = 998). 59.1% of participants had a poor Breslow’s seven health practice scores (BHPS), 32.9% had a moderate score, and 8.0% had a good score. Multivariable logistic regression analysis revealed that higher BHPS had a significantly higher prevalence of food stockpiling. Additionally, the interrupted group had the highest percentage of participants with low BHPS. Lower BHPS was significantly associated with interrupted stockpiled in the adjusted models. Among the seven health practices, the odds ratio of the “eating breakfast” practice was high. There was a significant positive association between higher health practice scores and food stockpiling for disasters in areas with a high risk of food shortage due to the predicted earthquake. Particularly, it was clarified that individuals who had fewer good health practices were associated with ending up interrupting food stockpiling.

## 1. Introduction

In recent years, natural disasters such as earthquakes and hurricanes have occurred frequently all over the world [1]. Great disasters have been predicted to occur in Japan, a country prone to natural disasters, in the near future. The Headquarters for Earthquake Research Promotion statistically processes past seismic activity records found from historical records and research studies and publishes long-term evaluation results for earthquakes [2]. The greatest predicted disaster is the Nankai Trough earthquake [2]. The Nankai Trough, marking the boundary between the Eurasian Plate and the Philippine Sea Plate, is forecasted to create a catastrophic earthquake underneath the Pacific Ocean off the coast of Japan from Shizuoka to Kyushu.

In the predicted disaster area, a large food shortage is estimated by the Japanese government (about 32 million meals in total for three days after the disaster) [3]. Especially Kochi, Tokushima, Wakayama, Ehime, and Mie are the top five prefectures (prefecture is a local government with wide-area and comprehending city, town, and village) with the highest estimated number of evacuees per population [4], and the risk of food shortage is expected to be high. Such a food shortage is due to the collapse of the food supply chain and logistics [5]. Additionally, people choose the individualistic strategy such as panic buying and overbuying by the perception of lacking food in the household. Hence, as a preparation for this emergency, it is important to stockpile food for each household before the event of a disaster.

The factors associated with stockpiling for disasters (e.g., emergency kits) have been clarified in previous studies. For example, the associated factors were advanced age, male sex, higher income, and higher educational background [6,7,8,9,10] (i.e., socioeconomic status (SES) factors). However, although Japan has experienced many large disasters in the last 10 years, the improvement rate from 2011 (the year of the Great East Japan Earthquake) is only 6.4% [11,12] because there are many households that have never stockpiled food and also those whose stockpiling has been interrupted. Indeed, 46.2% of the households had no food stockpiled for potential disasters in Japan in 2019 [11]. Especially in areas with expected detrimental damage after the Nankai Trough earthquake, the food stockpile rate is even lower than the national average [11]. Therefore, it is an urgent issue that each household promotes the accumulation of food for disasters in the predicted disaster areas. Improving food stockpiling requires an approach from a different perspective. It is considered that there is a lack of study to clarify the factors that can be improved by education or intervention even if the influence of SES factors associated with food stockpiling is adjusted.

A previous Japanese study showed that food stockpiles were treated as luxury goods [13]. Luxury goods are often refrained from being purchased, but the situation may change when people recognize association with such as health promotion. An example is a functional food. It is also perceived as a luxury good [14]. Functional foods are consumed by people with a healthy lifestyle [15]. Food stockpiling may also be possible to consider as same as functional foods. In other words, even if food stockpiling was perceived as luxury goods, recognition of the need to maintain people’s lives and health may lead to promote behavior of food stockpiling. Indeed, food stockpiling to prepare for a disaster is helpful to maintain individuals’ own and their family’s life and health in the event of a disaster [16]. The recognition that food stockpiling is helpful to maintain individuals’ own and their family’s life and health may be associated with people’s healthy lifestyles or health practices. However, the association between their health practices and food stockpiling for disasters is unknown.

The present study aimed to investigate the association between health practices and food stockpiling for disasters in areas with a high risk of food shortage due to the Nankai Trough earthquake. Additionally, we clarified whether individuals who can start stockpiling or who end up interrupting it exhibit features of health practices.

## 2. Methods

### 2.1. Design and Data Collection

The present study used a cross-sectional design, collecting data via an online questionnaire. Recruitment for the survey sought 1200 Japanese individuals aged ≥20 years that were registered with an online survey company (Rakuten Insight, Inc.; a total 2.2 million registrants). When the number of participants exceeded our target of 1200 individuals, the company ceased recruitment. The survey was open between 18 December and 20 December 2019. In addition, the company paid some financial incentives (Rakuten Points) for participating.

The study populations were the top five prefectures in Japan that estimated food shortages after the Nankai Trough earthquake, calculated from the estimated number of evacuees [3] by the Japanese government for each prefecture’s population [4]. The five prefectures were Kochi, Tokushima, Wakayama, Ehime, and Mie. The target individuals were people who prepared meals in the family because it was assumed that they would also stock foods for disaster for the family. In a screening question that asked “Who in your family mainly prepares meals?—myself, someone other than myself, or do not eat at home”, those who answered “myself” were included in the survey.

### 2.2. Questionnaire

The questionnaire consisted of 35 items and was self-administered using computers or smartphones. This study used and analyzed 12 of the 35 questionnaire items. The 12 questionnaire items had three parts as shown below:

#### 2.2.1. Sociodemographic Information of Individuals

This part included questions on participants’ individual sex, age, employment status, educational background, disaster experience, and community activities. The community activities were defined as “participate” if participants answer “regularly” and “sometimes” for the question “How often have you participated in community activities in the past year?” Additionally, it also included participants’ family composition, household income, prefecture, and vulnerable people in the family. The questions that defined vulnerable people were: “Do you have vulnerable people in a disaster in your family? Please select all that apply—none, infant, child, pregnant, disabled person, elderly person, person with a chronic disease, person with any food allergies, person who do not speak Japanese, person with pressure ulcers, others.”

#### 2.2.2. Health Practices (Exposure)

Health practices were assessed by Breslow’s seven health practice scores (BHPS) [17], which are associated with mortality [18]. In determining the scale used in this study, we reviewed previous studies. First, a literature search was conducted in PubMed and J-STAGE using the following keywords: “scale” AND “health practices”, “healthy lifestyle”, and so on. Next, previous studies for non-Japanese populations was excluded. Additionally, the scale was adopted by following reasons: (1) it is a global standard scale, (2) it can be measured with few questions, and (3) its validity had been examined the questionnaire of Japanese version. As a result of reviewing various scales, BHPS was adopted as only scale that satisfies the above three requirements. As the example not adopted, the measure by Li et al. [19] was one of the scales that can be measured health practices with the five questions. However, it is not a global standard and its validity in Japanese has not examined. The Japanese Health Practice Index [20] is a scale that is measured with ten questions and its validity is being examined in Japanese. However, it is not a global standard scale. BHPS has validated in Japanese [21,22] and is widely used in recent years to assess health practice variables in Japan and other countries [23,24]. Additionally, the previous study suggests that modifying behavior (as measured with the BHPS) change other healthy behavior. A literature showed that individuals with higher BHPS were significantly associated with their dental health behavior, such as periodic dental health examination [25]. Thus, it may be useful to examine the association between BHPS and other health behavior. The BHPS measures respondents’ adherence to seven good health practices: never smoking cigarettes, regular physical activity, moderate or no use of alcohol, 7–8 h of sleep/day regularly, eating breakfast, and not eating between meals. Participates answered yes or no for these seven items. “Yes” responses were summed to provide a cumulative BHPS ranging from 0 to 7, which we categorized into three groups: 0–3, 4–5 and 6–7, just as in the original study [18].

#### 2.2.3. Stage of Food Stockpiling for Disaster (Outcome Measures)

**Figure 1:**
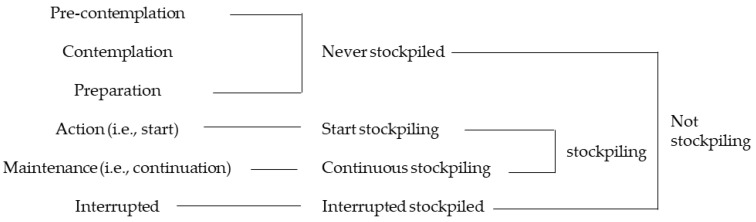
Stage of food stockpiling for disaster (outcome measure).

Stage of food stockpiling for disaster was assessed based on the transtheoretical model (TTM), which is a model derived from smoking cessation studies [26,27,28]. TTM is known to be useful for understanding the processes of change in diet-related behaviors [29]. The question is “Please select the item that applies to your status of food stockpiling for disaster at home”. The six answer items are as follows: (1) Pre-contemplation: not interested in stockpiling of food for disaster, (2) Contemplation: intending to stockpile food for disaster in the next six months, (3) Preparation: ready to stockpile food for disaster in the following month, (4) Action (i.e., start): stockpiled food for disaster but have not replaced it, (5) Maintenance (i.e., continuation): stockpiled food for disaster and have replaced it more than once, (6) Interrupted: previously stockpiled but not now. Because the ratio of the preparation stage was only 3.3% of the respondents, we combined the stages of “pre-contemplation”, “contemplation” and “preparation” as “never stockpiled”. As a result of this category compression, to describe other stockpiling status in an easy-to-understand, the “action” stage was classified as “start stockpiling” and “maintenance” as “continuous stockpiling” (Figure 1). “Never stockpiled” group defined people who had never stockpiled food before. “Start stockpiled” defined people who have stockpiled food once but have never purchased food that has expired. “Continuous stockpiling” defined people who have repurchased stockpile food that has expired and are still continuing. “Interrupted stockpiled” defined people who used to stockpile food but no longer do so and have interrupted the stockpiling.

### 2.3. Statistical Analysis

We applied logistic regression analysis to evaluate the strength of the association between the stage of food stockpiling for disaster and health practices.

First, we conducted a logistic regression analysis with food stockpiling as the dependent variable and SES factors as the independent variable. Results were presented as crude or adjusted odds ratios (ORs) with 95% confidence intervals (CI). We initially evaluated the variables through a univariable analysis and then performed a multivariable analysis (forced entry method) to adjust for all SES factors. A total of 998 responses were inputted into the logistic regression model, after excluding responses from participants whose responses to sex and educational background were “the others” and household income was “unknown” (*n* = 202).

Second, we conducted logistic regression analysis with food stockpiling as the dependent variable and BHPS and seven items of Breslow’s health practices as the independent variable. In addition, BHPS showed *p*-values for trend. We initially evaluated the variables through a univariable analysis and then performed a multivariable analysis (forced entry method) to adjust for some factors. Model 1 included the basic factors: sex (male or female) and age (20–34, 35–59, and ≥60 years). In addition to Model 2, the associated socioeconomic factors with food stockpiling in Table 2: educational background (below undergraduate, or above college degrees) were included. In Model 3, the community activities (participate or do not participate) were added, which is the associated modifiable factor with stockpiling [30].

Finally, we ascertained whether health practices affect behavior regarding food stockpiling (“start” or “interrupted”). First, “start stockpiling” was selected as the dependent variable (reference category: “never stockpiled”). Thereafter, “interrupted stockpiled” was selected (reference category: “continuous stockpiling”). The independent variable was the BHPS and seven items of Breslow’s health practices. The adjusted factors were the factors associated with food stockpiling, which is the same as in the second logistic regression analysis.

The *p*-values were two-sided, with *p* < 0.05 considered statistically significant. All statistical analyses were performed using IBM SPSS Statistics version 26.

## 3. Results

### 3.1. Association between SES Factors and Food Stockpiling

The participants’ characteristics are presented in Table 1 (*n* = 998). More females than males responded (66.6% vs. 33.4%). Table 1 shows participants’ characteristics according to the status of food stockpiling for disasters. A total of 40.0% (*n* = 399) of participants had stockpiled food for disaster.

The ORs for food stockpiling associated with SES factors are presented in Table 2. In the crude model, food stockpiling was significantly associated with seven SES factors. After adjusting for all variables in the model, the associated SES factors were sex (OR: 1.93, 95% CI: 1.37−2.71, *p* < 0.001), age (OR: 1.62, 95% CI: 1.34−1.96, *p* for trend < 0.001), and educational background (OR: 1.66, 95% CI: 1.26−2.19, *p* < 0.001).

### 3.2. Association between Health Practices and Food Stockpiling

Table 1 shows participants’ distribution of BHPS and those who had Breslow’s seven health practices, by food stockpiling status. A total of 59.1% of participants had a poor BHPS (0–3), 32.9% had a moderate score (4–5) and only 8.0% had a good score (6–7). Regarding each of the seven health practices, never smoking cigarettes was highest (71.1%). Following that, 68.3% of participants had a practice of eating breakfast. Many participants with a higher BHPS had stockpiled food. Furthermore, participants with good health practices had a high percentage of stockpiling food, regarding all seven health practices.

The ORs for food stockpiling associated with health practices are presented in Table 3. Higher BHPS had a significantly higher prevalence of food stockpiling (*p* for trend <0.001). This significant relationship persisted after adjustment for the associated factors of food stockpiling. As shown in Model 3, compared with 0–3 BHPS, 4–5 BHPS were 1.54 times more likely to have food stockpiling (adjusted OR: 1.54, 95% CI: 1.15–2.06), while 6–7 BHPS were 2.35 times more likely to have food stockpiling (adjusted OR: 2.35, 95% CI: 1.44–3.85). Among the items of Breslow’s seven health practices, five good health practices were significantly associated with food stockpiling even after adjusting for factors in Model 3. The practice most strongly associated with food stockpiling was “eating breakfast” (adjusted OR: 1.76, 95% CI: 1.31–2.37 in Model 3).

We evaluated interactions between SES factors and health practices by including interaction terms along with main effect terms. None of the interactions we tested were significant, even with *p* < 0.20 considered statistically significant.

### 3.3. Association between Health Practices and Food Stockpiling Stage (“Start” or “Interrupted”)

Table 4 shows participants’ distribution of BHPS and items of Breslow’s seven health practices by food stockpiling stage. Among of 998 participants, 466 participants were in “never stockpiled” group (*n* = 288 were “pre-contemplation” stage, *n* = 145 were “contemplation” stage, and *n* = 33 were “preparation” stage). Participants with a higher BHPS were part of the higher percentages of the “start” and “continuous” groups. The “interrupted” group had the highest percentage of participants with low BHPS.

The ORs for starting food stockpiles associated with health practices are presented in Table 5. Higher BHPS was significantly associated with start stockpiling (*p* for trend = 0.006) in Model 3. As shown in Model 3, compared with 0–3 BHPS, 4–5 BHPS were 1.53 times more likely to start food stockpiles (adjusted OR: 1.53, 95% CI: 1.02–2.30), while 6–7 BHPS were 2.26 times more likely to start food stockpiles (adjusted OR: 2.26, 95% CI: 1.11–4.61). Among the items of Breslow’s seven health practices, the highest OR for starting stockpile was “eating breakfast”: adjusted OR (95% CI) was 1.71 (1.16–2.62) in Model 3.

The ORs for interrupting food stockpiles associated with health practices are presented in Table 6. Lower BHPS was significantly associated with interrupted stockpiled in all models. Individuals having 4–5 and 6–7 BHPS were less likely to interrupted stockpiled food than those who have 0–3 BHPS: adjusted ORs (95% CI) in Model 3 were 0.47 (0.29–0.79) and 0.38 (0.16–0.87), respectively.

## 4. Discussion

The present study investigated the association of health practices with food stockpiling for disasters at home using an online survey. The results showed that there was a significant positive association between higher health practice scores and food stockpiling for disasters in areas with a high risk of food shortage due to the predicted Nankai Trough earthquake. In particular, it was clarified that individuals who had poor health practices may be at risk of interrupting food stockpiling.

### 4.1. Association between SES Factors and Food Stockpiling

Our results contributed to clarifying the SES factors associated with food stockpiling. The associated SES factors were sex, age, and educational background in the adjusted model. Contrary to expectations, household income was not significant in the adjusted model. Interestingly, household income was positively associated with stockpiling (in crude model), while employed individuals were negatively associated. One reason for this is that our survey targeted individuals were who prepare meals in the family. Hence, it is possible that the individuals in the family who mainly earn income and those who prepare meals were different.

### 4.2. Association between Health Practices and Food Stockpiling

Previous studies reported that having one health practice causes other health practices at the same time or in succession [31,32]. This study showed that individuals with higher BHPS (i.e., having a lot of good health practices) have stockpiled food for disasters. Indeed, many papers reported that survivors experienced an increased risk of health problems after disaster [33,34,35,36,37,38,39,40]. Because individuals with many good health practices consider not only their daily health but also their health after a disaster, they may have been associated with food stockpiling. There is literature showing that food stockpiling behavior was promoted by a set of multiple motivations and subjective risk perception during the COVID-19 pandemic [41]. Food stockpiling was treated as luxury goods [13]. Hence, further studies are needed on the factors that promote stockpiling behavior, but it cannot be denied that health practice may be the factor that promotes stockpiling.

Particularly, our finding found that attention should be paid to individuals whose stockpiling was interrupted. As we analyzed the stage of food stockpiling in detail, lower BHPS (i.e., having fewer good health practices) were associated with interrupting food stockpiling. Maintaining health practices was associated with higher self-efficacy [42]. Self-efficacy was led by performance accomplishments [43]. Therefore, in order to increase the self-efficacy of health practices, it is important to obtain a sense of accomplishment by starting one health practice. Although the causal relationship between the outcome and exposure is unclear in this study design, it may be possible that increasing good health practices may lead to stockpiling food for saving their life in disasters. A previous study indicate that one health practice could be a possible “gateway” behavior toward their behavior for saving their own life (i.e., seat belt use) [31]. Hence, proposing an approach that gives a gateway to increase health practices may be useful for continuing food stockpiles.

As a gateway to increase good health practices, it may be that education or intervention to have the practice of “eating breakfast” may be a good approach. Interestingly, our results showed that “eating breakfast” had the greatest association with food stockpiling. Since the clarified factors associated with the stockpile in the previous studies were mainly SES factors, this finding is important. The breakfast skippers paid less attention to health and had less nutrition knowledge [44]. For breakfast-skippers, nutrition promotion programs helped improve not only breakfast skipping but also various eating behaviors [45]. Therefore, implementing nutrition promotion programs for breakfast skippers may also positively associate promotion of food stockpiling. In addition, further effects can be expected by including content about food stockpiling for nutrition promotion programs. The findings of the effectiveness of simultaneous interventions in multiple health behaviors [46,47] would also support this consideration.

Additionally, not only eating breakfast but also “weight maintenance” was positively associated with start stockpiling among the items of Breslow’s seven health practices. Individuals who did not have good practice around “sleep,” “drinking”, and “snacking” were likely to interrupt stockpiling food. As shown in Table 4, it was found that the interrupted group had a lower proportion of people who have these three good health practices compared other groups. A previous study found an association between sleep and drinking [48,49]. Therefore, it can also be surmised that these practices may influence each other. To increase these health practices, national policies based on “health promotion” may be effective. For example, healthy people in the United States [50] aim to improve their health habits, such as sleep and drinking, which were suggested to be important in this study. Additionally, it may be expected that professionals such as registered dietitians and public health nurses working on these improvements of health practices will also promote food stockpiling by considering disasters.

### 4.3. Limitations of This Study

Several limitations of this study should be acknowledged. First, the study participants were recruited from monitors of an online survey company, and the study was conducted in one specific area. Accordingly, the generalizability of the results might be limited because of sampling bias. However, it was confirmed that the distribution of household income of this survey area’s population and participants are similar according to the national survey [51]. Second, the questionnaire did not examine the validity of online surveys. In addition, although the questionnaire included items based on TTM, further study is needed to verify whether this modification is appropriate. Third, the study participants were limited to only those who prepared meals in the family. The act of stockpiling food is affected everyone in the family. Further consideration is needed to determine whether the selection of survey targets is appropriate because risk factors could change depending on who in the family is stockpiling. It was also a limitation of this study that the health, age, and number of family members in the household were not accounted for. Fourth, a definition of food stockpiling was not clear in this survey. Because we left definition of stockpiling to the respondents with the question, “Please select the item that applies to your status of food stockpiling for disaster at home.” In the future, a survey with a clear definition of stockpiling is necessary. Finally, given that our study was cross-sectional in design, we were unable to confirm the existence of causal relationships. In contrast, there were two advantages to using an online survey: (1) because it was conducted using an online survey company, there was no bias due to the influence of the researcher, and (2) there was no substitute response (it is highly possible that we could obtain responses from the study target, which referred to people who prepared meals for the family).

## 5. Conclusions

Having more good health practices had a significant association with food stockpiling for disasters in areas with a high risk of food shortages due to the predicted Nankai Trough earthquake. In particular, it was clarified that individuals who had fewer good health practices were associated with interrupting food stockpiling. Although the causal relationship between the outcome and exposure is unclear in this study design, it may be possible that increasing good health practices could help to promote stockpiling food for disasters. In order to improve individuals’ health practices, it will be expected education and intervention by specialists such as registered dietitians and public health nurses.

## Figures and Tables

**Table 1:** Participants’ characteristics according to the status of food stockpiling for disasters (*n* = 998).

	All Participants (*n* = 998)		The Status of Food Stockpiling for Disaster			
			Not Stockpiling (*n* = 599)		Stockpiling (*n* = 399)	
Participants’ Characteristics	*n*	(%)	*n*	(%)	*n*	(%)
**Sex**						
Male	333	(33.4)	225	(37.6)	108	(27.1)
Female	665	(66.6)	374	(62.4)	291	(72.9)
**Age**						
20–34 years	311	(31.2)	203	(33.9)	108	(27.1)
35–59 years	497	(49.8)	306	(51.1)	191	(47.9)
≥60 years	190	(19.0)	90	(15.0)	100	(25.1)
**Employment status**						
Unemployed	312	(31.3)	166	(27.7)	146	(36.6)
Employed	686	(68.7)	433	(72.3)	253	(63.4)
**Educational background**						
Below undergraduate	597	(59.8)	378	(63.1)	219	(54.9)
Above college degrees	401	(40.2)	221	(36.9)	180	(45.1)
**Disaster experience**						
None	808	(81.0)	494	(82.5)	314	(78.7)
Have experience	190	(19.0)	105	(17.5)	85	(21.3)
**Family composition**						
Single household	316	(31.7)	222	(37.1)	94	(23.6)
Others	682	(68.3)	377	(62.9)	305	(76.4)
**Household income**						
<6 million yen	714	(71.5)	446	(74.5)	268	(67.2)
≥6 million yen	284	(28.5)	153	(25.5)	131	(32.8)
**Prefecture**						
Mie pref.	294	(29.5)	174	(29.0)	120	(30.1)
Wakayama pref.	185	(18.5)	106	(17.7)	79	(19.8)
Tokushima pref.	146	(14.6)	86	(14.4)	60	(15.0)
Ehime pref.	277	(27.8)	172	(28.7)	105	(26.3)
Kochi pref.	96	(9.6)	61	(10.2)	35	(8.8)
**Vulnerable people in family**						
None	738	(73.9)	460	(76.8)	278	(69.7)
Presence	260	(26.1)	139	(23.2)	121	(30.3)
**Community activities**						
Do not participate	674	(67.5)	451	(75.3)	223	(55.9)
Participate	324	(32.5)	148	(24.7)	176	(44.1)
**BHPS (Breslow’s health practice score)**						
0–3	590	(59.1)	390	(65.1)	200	(50.1)
4–5	328	(32.9)	175	(29.2)	153	(38.3)
6–7	80	(8.0)	34	(5.7)	46	(11.5)
**Items of Breslow’s seven health practices ^†^**						
Never smoking cigarettes	710	(71.1)	412	(68.8)	298	(74.7)
Eating breakfast	682	(68.3)	375	(62.6)	307	(76.9)
7–8 h sleep/day regularly	450	(45.1)	257	(42.9)	193	(48.4)
Moderate or no use of alcohol	441	(44.2)	236	(39.4)	205	(51.4)
Maintaining proper weight	389	(39.0)	200	(33.4)	189	(47.4)
Regular physical activity	353	(35.4)	189	(31.6)	164	(41.1)
Not eating between meals	120	(12.0)	60	(10.0)	60	(15.0)

^†^ Items of Breslow’s seven health practices are shown in the number and percentage of participant who have a practice in each.

**Table 2:** Statistical association between SES factors and food stockpiling for disaster (*n* = 998).

	Crude Model			Adjusted Model ^†^		
Variables	OR	(95% CI)	*p* for Trend	OR	(95% CI)	*p* for Trend
**Sex**						
Male	Ref			Ref		
Female	1.62	(1.23–2.14) **		1.93	(1.37–2.71) ***	
**Age**			1.39 (1.17–1.64) ***			1.62 (1.34–1.96) ***
20–34 years	Ref			Ref		
35–59 years	1.17	(0.87–1.58)		1.43	(1.04–1.97) *	
≥60 years	2.09	(1.45–3.02) ***		2.96	(1.93–4.53) ***	
**Employment status**						
Unemployed	Ref			Ref		
Employed	0.66	(0.51–0.87) **		0.88	(0.64–1.19)	
**Educational background**						
Below undergraduate	Ref			Ref		
Above college degrees	1.41	(1.09–1.82) *		1.66	(1.26–2.19) ***	
**Disaster experience**						
None	Ref			Ref		
Have experience	1.27	(0.93–1.75)		1.35	(0.96–1.88)	
**Family composition**						
Single household	Ref			Ref		
Others	1.91	(1.44–2.54) ***		1.39	(0.99–1.96)	
**Household income**						
<6 million yen	Ref			Ref		
≥6 million yen	1.43	(1.08–1.88) *		1.30	(0.96–1.76)	
**Prefecture**						
Mie pref.	Ref			Ref		
Wakayama pref.	1.08	(0.74–1.57)		0.98	(0.66–1.44)	
Tokushima pref.	1.01	(0.68–1.51)		0.99	(0.65–1.50)	
Ehime pref.	0.89	(0.63–1.24)		0.83	(0.59–1.18)	
Kochi pref.	0.83	(0.52–1.34)		0.87	(0.53–1.44)	
**Vulnerable people in family**						
None	Ref			Ref		
Presence	1.44	(1.08–1.92) *		1.31	(0.94–1.83)	

^†^ All variables in model are listed in the table. OR: odds ratio, CI: confidence intervals. *** *p* < 0.001, ** *p* < 0.01, * *p* < 0.05.

**Table 3:** Statistical association between health practices and food stockpiling for disaster (*n* = 998).

	Crude Model	Model 1	Model 2	Model 3
Variables	OR (95% CI)	OR (95% CI)	OR (95% CI)	OR (95% CI)
**BHPS ^†^**	*p* for trend < 0.001	*p* for trend < 0.001	*p* for trend < 0.001	*p* for trend < 0.001
0–3	Reference	Reference	Reference	Reference
4–5	1.71 (1.29–2.25) ***	1.63 (1.23–2.16) **	1.55 (1.17–2.06) **	1.54 (1.15–2.06) **
6–7	2.64 (1.64–4.24) ***	2.45 (1.51–3.97) ***	2.35 (1.45–3.83) **	2.35 (1.44–3.85) **
**Items of Breslow’s seven health practices**				
Never smoking cigarettes	1.34 (1.01–1.78) *	1.27 (0.95–1.70)	1.20 (0.90–1.61)	1.18 (0.88–1.59)
Eating breakfast	1.99 (1.50–2.65) ***	1.81 (1.36–2.43) ***	1.77 (1.32–2.38) ***	1.76 (1.31–2.37) ***
7–8 h sleep/day regularly	1.25 (0.97–1.61)	1.24 (0.96–1.61)	1.24 (0.96–1.62)	1.19 (0.91–1.56)
Moderate or no use of alcohol	1.63 (1.26–2.10) ***	1.55 (1.20–2.02) **	1.52 (1.17–1.98) **	1.47 (1.12–1.91) **
Maintaining proper weight	1.80 (1.39–2.33) ***	1.66 (1.27–2.16) ***	1.61 (1.24–2.11) ***	1.55 (1.18–2.04) **
Regular physical activity	1.51 (1.16–1.97) **	1.54 (1.17–2.02) **	1.49 (1.13–1.96) **	1.46 (1.11–1.93) **
Not eating between meals	1.59 (1.08–2.33) *	1.70 (1.14–2.54) **	1.68 (1.12–2.50) **	1.73 (1.15–2.60) **

^†^ Breslow’s health practice score. In addition to Model 1, sex and age were included. In addition to Model 2, sex, age, and educational background were included. In addition to Model 3, sex, age, educational background, and community activities were included. *** *p* < 0.001, ** *p* < 0.01, * *p* < 0.05. OR: odds ratio, CI: confidence intervals.

**Table 4:** The distribution of BHPS and items of Breslow’s seven health practices by food stockpiling stage (*n* = 998).

	The Stage of Food Stockpiling for Disaster							
	“Never” vs. “Start”				“Continuous” vs. “Interrupted”			
	Never Stockpiled (*n* = 466)		Start Stockpiling (*n* = 158)		Continuous Stockpiling (*n* = 241)		Interrupted Stockpiled (*n* = 133)	
	*n*	(%)	*n*	(%)	*n*	(%)	*n*	(%)
**BHPS (Breslow’s health practice score)**								
0–3	295	(63.3)	76	(48.1)	124	(51.5)	95	(71.4)
4–5	145	(31.1)	67	(42.4)	86	(35.7)	30	(22.6)
6–7	26	(5.6)	15	(9.5)	31	(12.9)	8	(6.0)
**Items of Breslow’s seven health practices ^†^**								
Never smoking cigarettes	326	(70.0)	123	(77.8)	175	(72.6)	86	(64.7)
Eating breakfast	293	(62.9)	120	(75.9)	187	(77.6)	82	(61.7)
7–8 h sleep/day regularly	206	(44.2)	75	(47.5)	118	(49.0)	51	(38.3)
Moderate or no use of alcohol	188	(40.3)	82	(51.9)	123	(51.0)	48	(36.1)
Maintaining proper weight	152	(32.6)	77	(48.7)	112	(46.5)	48	(36.1)
Regular physical activity	147	(31.5)	56	(35.4)	108	(44.8)	42	(31.6)
Not eating between meals	49	(10.5)	20	(12.7)	40	(16.6)	11	(8.3)

^†^ Items of Breslow’s seven health practices are shown in the number and percentage of participant who have a practice in each.

**Table 5:** Statistical association between “start stockpiling” and health practices (*n* = 624) ^†^.

	Crude	Model 1	Model 2	Model 3
Variables	OR (95% CI)	OR (95% CI)	OR (95% CI)	OR (95% CI)
**BHPS** ^‡^	*p* for trend = 0.001	*p* for trend = 0.003	*p* for trend = 0.006	*p* for trend = 0.006
0–3	Reference	Reference	Reference	Reference
4–5	1.79 (1.22–2.63) **	1.67 (1.13–2.47) *	1.55 (1.05–2.31) *	1.53 (1.02–2.30) *
6–7	2.24 (1.13–4.44) *	2.16 (1.07–4.33) *	2.15 (1.06–4.32) *	2.26 (1.11–4.61) *
**Items of Breslow’s seven health practices**				
Never smoking cigarettes	1.51 (0.99–2.31)	1.40 (0.91–2.15)	1.32 (0.85–2.04)	1.30 (0.83–2.02)
Eating breakfast	1.87 (1.24–2.81) **	1.74 (1.15–2.65) **	1.70 (1.12–2.59) *	1.71 (1.16–2.62) *
7–8 h sleep/day regularly	1.14 (0.79–1.64)	1.10 (0.76–1.60)	1.13 (0.78–1.65)	1.04 (0.71–1.52)
Moderate or no use of alcohol	1.60 (1.11–2.30) *	1.48 (1.02–2.14) *	1.44 (0.99–2.09)	1.39 (0.95–2.03)
Maintaining proper weight	1.96 (1.36–2.84) ***	1.76 (1.21–2.55) **	1.69 (1.16–2.47) **	1.60 (1.09–2.35) *
Regular physical activity	1.19 (0.82–1.74)	1.29 (0.87–1.91)	1.25 (0.84–1.85)	1.24 (0.83–1.86)
Not eating between meals	1.23 (0.71–2.15)	1.34 (0.75–2.38)	1.34 (0.75–2.39)	1.33 (0.74–2.40)

^†^ Reference category of the dependent variable is “never stockpiled” (*n* = 466). ^‡^ Breslow’s health practice score. In addition to Model 1, sex and age were included. In addition to Model 2, sex, age, and educational background were included. In addition to Model 3, sex, age, educational background, and community activities were included. *** *p* < 0.001, ** *p* < 0.01, * *p* < 0.05. OR: odds ratio, CI: confidence intervals.

**Table 6:** Statistical association between “interrupted stockpiled” and health practices (*n* = 374) ^†^.

	Crude	Model 1	Model 2	Model 3
	OR (95% CI)	OR (95% CI)	OR (95% CI)	OR (95% CI)
**BHPS** ^‡^	*p* for trend < 0.001	*p* for trend < 0.001	*p* for trend = 0.001	*p* for trend = 0.001
0–3	Reference	Reference	Reference	Reference
4–5	0.46 (0.28–0.75) **	0.46 (0.28–0.75) **	0.47 (0.28–0.77) **	0.47 (0.29–0.79) **
6–7	0.34 (0.15–0.77) **	0.35 (0.15–0.80) *	0.37 (0.16–0.85) *	0.38 (0.16–0.87) *
**Items of Breslow’s seven health practices**				
Never smoking cigarettes	0.69 (0.44–1.09)	0.69 (0.44–1.10)	0.73 (0.46–1.16)	0.75 (0.47–1.21)
Eating breakfast	0.46 (0.29–0.74) **	0.48 (0.30–0.77) **	0.49 (0.30–0.79) **	0.50 (0.31–0.80) **
7–8 h sleep/day regularly	0.65 (0.42–0.998) *	0.60 (0.38–0.93) *	0.61 (0.39–0.96) *	0.61 (0.39–0.96) *
Moderate or no use of alcohol	0.54 (0.35–0.84) **	0.54 (0.35–0.84) **	0.55 (0.35–0.86) **	0.57 (0.36–0.89) *
Maintaining proper weight	0.65 (0.42–1.01)	0.68 (0.44–1.06)	0.69 (0.45–1.08)	0.70 (0.45–1.10)
Regular physical activity	0.57 (0.36–0.89) **	0.62 (0.39–0.98) *	0.64 (0.40–1.01)	0.66 (0.41–1.04)
Not eating between meals	0.45 (0.22–0.92) *	0.46 (0.22–0.95) *	0.48 (0.23–0.99) *	0.45 (0.22–0.94) *

^†^ Reference category of the dependent variable is “continuous stockpiling” (*n* = 241). ^‡^ Breslow’s health practice score. In addition to Model 1, sex and age were included. In addition to Model 2, sex, age, and educational background were included. In addition to Model 3, sex, age, educational background, and community activities were included. ** *p* < 0.01, * *p* < 0.05. OR: odds ratio, CI: confidence intervals.
